# Ankles back in randomized controlled trial (ABrCt): braces versus neuromuscular exercises for the secondary prevention of ankle sprains. Design of a randomised controlled trial

**DOI:** 10.1186/1471-2474-12-210

**Published:** 2011-09-27

**Authors:** Kasper W Janssen, Willem van Mechelen, Evert ALM Verhagen

**Affiliations:** 1 EMGO Institute for Health and Care Research, Department of Public & Occupational Health, VU University Medical Center, Amsterdam, the Netherlands

## Abstract

**Background:**

Ankle sprains are the most common sports and physical activity related injury. There is extensive evidence that there is a twofold increased risk for injury recurrence for at least one year post injury. In up to 50% of all cases recurrences result in disability and lead to chronic pain or instability, requiring prolonged medical care. Therefore ankle sprain recurrence prevention in athletes is essential. This RCT evaluates the effect of the combined use of braces and neuromuscular training (e.g. proprioceptive training/sensorimotor training/balance training) against the individual use of either braces or neuromuscular training alone on ankle sprain recurrences, when applied to individual athletes after usual care.

**Methods/Design:**

This study was designed as three way randomized controlled trial with one year follow-up. Healthy individuals between 12 and 70 years of age, who were actively participating in sports and who had sustained a lateral ankle sprain in the two months prior to inclusion, were eligible for inclusion. After subjects had finished ankle sprain treatment by means of usual care, they were randomised to any of the three study groups. Subjects in group 1 received an eight week neuromuscular training program, subjects in group 2 received a sports brace to be worn during all sports activities for the duration of one year, and group 3 received a combination of the neuromuscular training program and a sports brace to be worn during all sports activities for the duration of eight weeks. Outcomes were assessed at baseline and every month for 12 months therafter. The primary outcome measure was incidence of ankle sprain recurrences. Secondary outcome measures included the direct and indirect costs of recurrent injury, the severity of recurrent injury, and the residual complaints during and after the intervention.

**Discussion:**

The ABrCt is the first randomized controlled trial to directly compare the secondary preventive effect of the combined use of braces and neuromuscular training, against the use of either braces or neuromuscular training as separate secondary preventive measures. This study expects to identify the most effective and cost-efficient secondary preventive measure for ankle sprains. The study results could lead to changes in the clinical guidelines on the prevention of ankle sprains, and they will become available in 2012.

**Trial registration:**

Netherlands Trial Register (NTR): NTR2157

## Background

The burden of ankle sprains in sports is high. Ankle sprains are the most common sports and physical activity (PA) related injury [1-4]. It has been estimated that about 25% of all injuries across all sports are ankle injuries. Of all ankle injuries, about 85% are acute lateral ankle sprains. As an example, in the Netherlands the most recent count of sports injuries showed that there is an estimated absolute number of 3, 5 million acute sports injuries each year in a sporting population of 11 million participants [4]. Of these a total of 600,000 are to the ankle, making the estimated annual number of sports related ankle sprains 510,000, of which half is (para-)medically treated. A recent Dutch study [5] showed that, disregarding the requirement of medical treatment, the mean total (direct and indirect) cost of one ankle sprain is approximately €360. This would give a rough estimate of the annual sports related ankle sprain costs being €184 million.

In addition to the sheer magnitude of the ankle sprain 'problem' in sports, there is extensive evidence that there is a twofold increased risk for injury recurrence for at least one year post injury [6-9]. In up to 50% of all cases recurrences result in disability and lead to chronic pain or instability, requiring prolonged medical care [10]. This increased ankle sprain recurrence risk has been found to exist even after completion of medical treatment [9,11,12]. Therefore, the advocation of braces and neuromuscular training (synonymous: proprioceptive training/sensorimotor training/balance training) after usual care is a justified part of treatment, and has a significant and important impact on a patient's current and future health, as well as future sports and physical activity participation.

A recent systematic review revealed that both braces as well as neuromuscular training have been proven equally effective for the secondary prevention of ankle sprains [13]. Both account for an approximated 50% overall reduction in recurrent ankle sprain rate. In contrast, individuals with no history of ankle sprains do not seem to benefit from these preventive measures. Hence, it can be concluded from the current literature that despite different preventive pathways taping, bracing and neuromuscular training are separately linked to a similar secondary preventive effect [13]. Based on these outcomes, in theory, a combination of an external prophylactic measure (tape or brace) with neuromuscular training is argued to achieve the best preventive outcomes with minimal burden for the athlete.

Therefore, the current three-way randomised controlled trial evaluates the (cost-) effectiveness of the combined use of braces and neuromuscular training against the individual use of either braces or neuromuscular training alone.

## Methods/Design

The CONSORT statement was followed to describe the design of this study [14]. This statement is a checklist intended to improve the quality of reports of randomized controlled trials.

### Study outline

**Figure 1:**
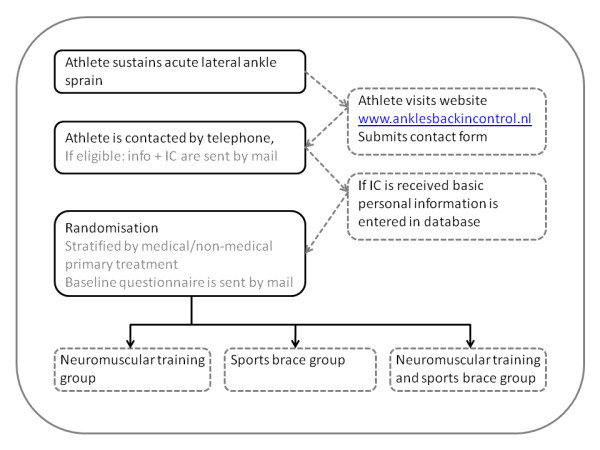
**Study design ABrCt and flow of the participants**.

The Ankles Back in randomised Controlled trial (ABrCt) is a three way randomized controlled trial with one year follow-up. The study design and flow of the participants are shown in Figure 1.

The study is funded by the Netherlands Organization for Health Research and Development (ZonMW). The study design, procedures and informed consent procedure were approved by the Medical Ethics Committee (number 31785.029.10) of the VU University Medical Centre, the Netherlands. Trial register number NTR 2157. All participants provided written informed consent.

### Hypotheses

The first hypothesis of this study is that each of the three interventions will lead to a 50% reduction of ankle sprain recurrence incidence. The second hypothesis is that there will be a difference in costs-effectiveness of the three interventions. The third hypothesis is that there will be differences in ankle sprain related complaints (e.g. chronic instability, pain, feeling of giving way) between the three interventions.

### Participants

Healthy participants between 18 and 70 years of age, who are actively participating in sports and who have sustained a lateral ankle sprain up to two months prior to inclusion, were eligible for inclusion in the study. All primary treatment options were allowed, i.e. no treatment, self treatment or (para-)medical treatment. Responders were excluded if they did not master the Dutch language, had a history of vestibular complaints, or had a different injury than a lateral ankle sprain in the same ankle (e.g. fracture of the ankle). This resulted in a diverse source population of athletes from all types and levels of sports participating in the study. The interventions were considered to be appropriate for all athletes. Hupperets et al found no side effects of the neuromuscular training program [15]. Other studies reported changed biomechanics, in the use of braces as a secondary preventive measures, but no other side effects were reported [16].

### Sample size

As there is no scientific evidence on the combined effect of braces and neuromuscular training it is assumed that the effectiveness is comparable to the separate effect of braces or neuromuscular training alone[13]. Therefore, while reliable effect-sizes to calculate power are missing, a power analysis was done from the view of the intervention costs.

A recent Dutch study on ankle sprains and their prevention collected direct and indirect cost data related to ankle sprains [5]. Within this data a subgroup analyses of previously injured athletes revealed that the mean costs of a recurrent ankle sprain are €27 with an SD of €108. Home based neuromuscular training program costs are estimated at €25. Sports braces costs are estimated at €50. Consequently usual care costs are about €75 per recurrent ankle sprain. Based on an expected difference of €50 in costs (i.e, the cost difference between usual care and a neuromuscular training program), a total of 99 participants per group is needed. Taken into account an attrition rate of 20% this means that a total sample of 356 participants is required at baseline.

### Recruitment of study population

Participants were recruited through the internet. Information on the study and a call for participation were available on the website: http://www.anklesbackincontrol.nl. The study was supported by the Dutch Association for Sports and Exercise Medicine (VSG), the Royal Dutch Physiotherapy Association (KNGF), the Dutch Orthopedic Association (NOV) and the Dutch College of General Practitioners (NHG). The participating organizations placed a hyperlink on their website. Hereby, individuals seeking medical information regarding ankle sprains in any of these sites came across this call for participants. Similar calls were placed on the websites of sporting associations of sports with a relatively high ankle sprain rate (e.g. soccer, volleyball, handball, basketball, korfball and tennis). Where possible existing electronic newsletters of sporting associations were used to contact potential participants directly. Next to the mentioned calls for participation on websites, we recruited actively by placing calls for participation on sports-related internet fora, as well as by electronic newsletters. This method of recruitment resulted in a sample of participants from a wide spectrum of different activities, sports and ages. Thus we created a sample representative to the Dutch sporting population. A similar recruitment strategy was successfully employed in a previous study on the same topic [15].

Recruitment of participants for the ABrCt study took place between April 2010 and May 2011. A total of 450 people were interested to participate in the ABrCt study and reacted on the call for subjects with a recent ankle sprain. A total of 18 athletes were classified as non-responders after a maximum of ten attempts to contact them through telephone or email failed. Of 432 people who were available for inclusion, 48 were excluded. Reasons for exclusion were: no return of baseline questionnaire (3), still undergoing treatment at the end of the inclusion period (5), no agreement on informed consent (2), vestibular complaints (2), non-recent ankle sprain (injury > 2 months before inclusion) (25), other serious injury (e.g. fracture of the ankle) (5) and private reasons (6). After completion of the baseline questionnaire and informed consent, 384 athletes were randomly assigned to the neuromuscular training group (n = 122), brace group (n = 126), and combined group (n = 136).

### Randomization procedure

After subjects had finished ankle sprain treatment by means of usual care, they were randomized to any of the three study groups. Stratification was based on type of usual care; (para-)medical treatment or no treatment/other treatment. Randomization took place at the end of usual care in order to minimize the chance of alterations in the treatment due to prior knowledge of the allocated intervention. As an example, a caregiver might decrease the amount of strengthening exercises when a patient was allocated to the neuromuscular training group. Even so, the other way around a caregiver might feel obliged to include additional strength and balance exercises when a patient is allocated to the brace group. Therefore, participants were asked to follow their usual treatment and/or rehabilitation program and received the allocated secondary preventive intervention after usual care had finished (and ideally before sports participation commenced).

### Interventions

The focus of this study is to evaluate the effectiveness and cost-effectiveness of secondary preventive measures after usual care for acute lateral ankle sprains. Therefore, after ankle sprain treatment and from the moment that sports participation commenced, participants received their allocated intervention protocol. Subjects in group 1 received an eight week neuromuscular training program, subjects in group 2 received a sports brace to be worn during all sports activities for the duration of one year, and group 3 received a combination of the neuromuscular training program and a sports brace to be worn during all sports activities for the duration of eight weeks.

**Figure 2:**
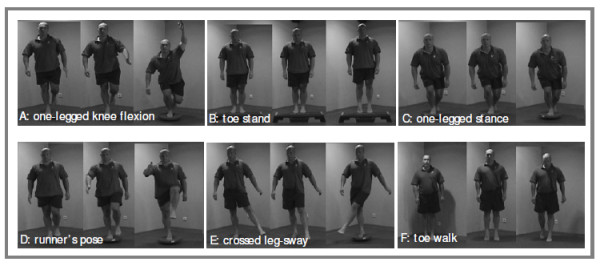
**Home based neuromuscular training program, exercises A to F**.

Subjects allocated to the neuromuscular training group received a standardized eight-week unsupervised home based neuromuscular training program (Figure 2). This program has recently been proven effective for the secondary prevention of ankle sprains within a comparable setting [15].

**Figure 3:**
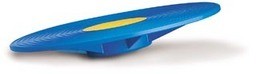
**Rock ankle exercise board, Avanco Sweden**.

All participants of the neuromuscular training group received the same balance board (Figure 3) for free. Furthermore general written and visual information on the duration and intensity of the program were provided. In addition, a website was created including basic information on the project and a section only accessible for each group's subjects. The exercise frequency was, with three training sessions per week, consistent throughout the full eight weeks (Table 1). The six exercises (A tot F) become more challenging as the program progressed (Table 2).

**Table 1:** Exercise schedule

**Exc**.	*Week 1*			*Week 2*			*Week 3*			*Week 4*			*Week 5*			*Week 6*			*Week 7*			*Week 8*		
	***1***	***2***	***3***	***4***	***5***	***6***	***7***	***8***	***9***	***10***	***11***	***12***	***13***	***14***	***15***	***16***	***17***	***18***	***19***	***20***	***21***	***22***	***23***	***24***

**A**	1	1	1	1	1	1	1	1	2	2	2	2	2	2	2	2	3	3	3	3	3	3	3	3
**B**	1	1	1	1	1	1	1	1	1	1	1	1	2	2	2	2	2	2	2	2	2	2	2	2
**C**	1	1	1	1	2	2	2	2	3	3	3	3	3	3	3	3	3	3	3	3	3	3	3	3
**D**	1	1	1	1	2	2	2	2	3	3	3	3	3	3	3	3	3	3	3	3	3	3	3	3
**E**	1	1	1	1	1	1	2	2	2	2	2	2	3	3	3	3	4	4	4	4	4	4	4	4
**F**	1	1	1	1	1	1	1	1	1	1	1	1	2	2	2	2	2	2	2	2	2	2	2	2

The content of each training session is shown vertically from session 1 to 24 (session numbers are printed bold). The exercises in each session are numbered A to F, the number (1 to 4) describes the difficulty level. Explanation of the difficulty level is given in Table 2.

**Table 2:** Exercise difficulty level

Exc.	Difficulty level	Exc.	Difficulty level
**A**	1. on even surface	**E**	1. on even surface; with handheld
	2. on even surface; eyes shut		2. on even surface; without handhold
	3. on balance board		3. on even surface; eyes shut and without handhold
**B**	1. on high surface; with handhold		4. on balance board
	2. on high surface; without handhold	**F**	1. on even surface; walking
**C**	Same 3 levels as exercise A		2. on even surface; jumping
**D**	Same 3 levels as exercise A		

**Figure 4:**
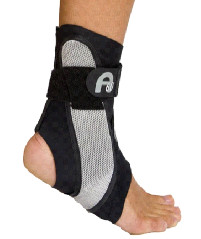
**Aircast A60 Ankle Support brace**.

Subjects allocated to the brace group received an Aircast A60 Ankle Support brace for free (Figure 4). This is a semi-rigid ankle brace, specifically designed for use during sports. The design incorporates a sleek stabilizer located on either side of the ankle. This stabilizer is molded at a 60 degree angle to help guard against ankle sprains and prevent rollover. The light-weight anatomic design fits in athletic footwear. The brace is applied and adjusted with a single strap that securely holds it in place. The A60 is available in three sizes: small, medium, and large, with left and right models to guarantee optimal fit. Participants were encouraged to wear this brace during all sports activities for the duration of the entire 12 months follow-up.

Subjects allocated to the combined group received both the standardized eight-week unsupervised home based neuromuscular training program and an Aircast A60 Ankle Support brace for free. Participants in this group were encouraged to wear the ankle brace during all sporting activities over a period of 8 weeks. The latter under the assumption that the neuromuscular program has achieved its preventive effect after 8 weeks, after which the use of a sports brace is considered redundant [13].

### Outcome measures

The primary outcome measure was incidence of ankle sprain recurrences, presented as the number of ankle sprain recurrences per 1,000 hours of sports participation. The following generic injury definition was used in this study: "An ankle sprain occurring as a result of sports participation or other daily activities and which causes one or more of the following:

• the subject has to stop the sports activity; and/or

• cannot (fully) participate in the next planned sports activity; and/or

• cannot go to work/school the next day; and/or

• needs medical attention (ranging from onsite care by e.g. first aid personnel, to personal care by e.g. physiotherapist, ER-doctor, or sports physician)."

Secondary outcome measures included the direct and indirect costs of recurrent injury, the severity of recurrent injury, and the residual complaints during and after the intervention.

This study has a one-year follow-up with measurements scheduled monthly for 12 months after baseline. All questionnaires and other forms were web-based and an invitation to fill out the questionnaires was sent to athletes by e-mail (with hyperlink), unless requested otherwise by the participant.

### Baseline measurement

When a potential participant responded to one of our calls, he or she was contacted by phone. During this initial contact the background and proceedings of the proposed study were explained by protocol. When the potential participant agreed to partake in this study an informed consent was sent by ordinary mail which he or she was required to sign and return by ordinary mail within one week. In addition, an e-mail was sent containing a hyperlink to the baseline questionnaire, which he or she was required to complete within one week. This baseline questionnaire gathered information of each participant on demographic variables, physical characteristics, sports & injury history, use of preventive measures, details and injury mechanisms of the current ankle sprain, and subsequent treatment and/or rehabilitation.

In order to create similar groups with respect to the outcome measures, participants were stratified by treatment, i.e. (para-)medical treatment or other/no treatment. As gender has never been identified as a factor related to secondary ankle sprain complaints, this was not a parameter for stratification. The participant was randomized to any of the three study groups and received oral and written instructions for the specific group allocation directly after completion of usual care.

### Follow-up measurement

Follow-up measurements commenced after randomization, and took take place once a month for a total period of 12 months. The monthly questionnaires gathered information on sports participation, on the use of preventive measures and sustained injuries to the lower extremities in the preceding month. Per training or match session the total minutes of participation were registered. Finally, the follow-up questionnaires measured residual complaints of the initial ankle sprain; pain, feeling of giving way and subjective restriction in range of motion of the ankle.

### Compliance

In a similar study compliance has been shown to significantly affect the effect outcomes of a preventive measure [17]. Therefore compliance to the allocated intervention protocol was measured through the monthly questionnaire. Participants in the neuromuscular training group were asked if they completed all (> 75%), most (more than 50%), a few (about 25%), or almost none of the prescribed training sessions during the 8 week intervention. Participants in the brace group were asked each month during follow-up whether they always (> 75%) wore the brace during sports, most of the time (more than 50%), a few times (about 25%), or almost none of the time during the prescribed period. Participants in the combination group were asked both questions during the 8 week intervention. After cessation of the prescribed intervention period participants were asked, as part of the monthly follow-up questionnaire, if they had performed additional neuromuscular training or wore a brace by own initiative.

### Injury registration

When a participant sustained an ankle injury during the follow-up period, the participant acknowledged this by e-mail. As a fail safe, ankle sprain recurrences were also recorded through the monthly follow-up questionnaire. After an injury was registered, the participant received an injury registration form. This form contained detailed questions on the diagnosis, the cause, and the aetiology of the re-injury. Furthermore, the advised treatment and the person who treated the injury were registered. The same form has been previously employed successfully in ankle sprain prevention studies [11,15]. Based on this form, a cost diary was sent to the athlete.

To evaluate the cost-effectiveness of the different intervention interventions participants who sustained an ankle sprain received a cost-diary.

The cost-diary is a log, which registered all absence from work, school and other chores of life, and (para-)medical treatment (including use of medication) from the moment of injury onwards until full recovery. From these cost-diaries direct and indirect costs resulting from the sustained ankle sprain could be calculated for use in an economic evaluation.

### Cost-effectiveness evaluation

The aim of the economic evaluation was to determine and compare the total costs for subjects in all three trial arms, and to relate these costs to the effects of these groups.

The economic evaluation was performed from a societal perspective. Table 3 provides an overview of the costs collected [18,19]. Cost of the intervention included costs that were directly related to the implementation of the intervention programmes. These costs included for the neuromuscular training program, the written information materials, an instructional video, development and maintenance of an informational website, and the balance boards. For the brace group only the costs of the brace and maintenance of an informational website were taken into account.

**Table 3:** Costs as applied in the economic evaluation

Costs	Cost (€)
Direct health care costs:	
General practitioner (mean cost of total visits)^	87.19
Physical therapist (per visit = 30 min)*	28.00
Sports physician (per visit = 30 min)*	80.00
Medical specialist (mean cost of total visits)#	89.24
Hospital costs (if seen by specialist)#	397.80
Alternative therapist (per visit)*	27.20
Emergency room (per visit) ^	197.12
Drugs‡	n.a.
Medical devices‡	
Tape (per roll)	4.00
Brace	79.50
Crutches (rent per week)	15.00
Indirect costs:	
Absenteeism from paid work (per day)§	n.a.
Absenteeism from unpaid work (per hour)*	7.94

€ 1.00 = $ 1.47 = £ 0.89 (d.d. 07-06-11)

* Costs according to Dutch guidelines

# Costs according to DBC (Zorgautoriteit 2011)

‡ According to tariff of the Royal Dutch Society of Pharmacy

§ Indirect costs for paid work was calculated for each injured separately based on mean income of the Dutch population according to age and sex

^ According to cost research on self-reference to GP and ER [22]

Besides the cost of the intervention itself, direct health care costs were included: i.e. costs of care by a general practitioner, physiotherapist, massage therapist, alternative therapist, and care by a sports physician or other medical specialist (e.g., orthopaedic surgeon, general surgeon); hospital care; use of drugs (e.g., paracetamol/actaminophen, ibuprofen) and medical devices (e.g., crutches, tape, braces). The costs of drugs were estimated on the basis of prices recommended by the Royal Dutch Society of Pharmacy [19]. Also indirect costs resulting from a loss of production due to absenteeism from paid or unpaid work were included. Indirect costs for absenteeism from paid work were calculated using the friction cost approach of 4 months, based on the mean age and sex-specific income of the Dutch population [18,20]. Indirect costs for productivity loss of unpaid work, such as study and household work, costs were estimated at a shadow price of €7.94 an hour [18].

### Process evaluation

A process evaluation was conducted for the three interventions. A supplement questionnaire was added to the monthly questionnaire after the intervention: for the neuromuscular training group after 2 months; for the brace group after 2 months and after 1 year; and for the combined group 2 questionnaires: one for evaluation of the training and one for evaluation of the brace, both added after 2 months. The questionnaire contained questions on the subjective response to the program (e.g. attitude towards neuromuscular training and braces), the presentation of the interventions, the perceived effect of the interventions, the support from peers for the participation in the program and questions on motivation for, and-compliance to the program.

### Statistical analyses

To evaluate the success of the randomisation, baseline values were analysed for differences between intervention group and control group, using a chi-square for categorical data and a student's t-test for numerical data.

All analyses were carried out according to the intention-to-treat principle. Cox-regression analysis was used to compare ankle recurrence risk between the intervention groups, using a significance level of P < 0.05. Other variables were checked for confounding and/or effect-modification and were adjusted for accordingly. Absence from sports was tested between groups using a Mann-Whitney test, since absence from sports due to an injury is not normally distributed [11].

Mean direct, indirect and total costs were estimated and compared between the three groups, both for the costs per subject in the injured population and for the costs per subject in the total population. Because costs are not normally distributed, 95% confidence intervals for the differences in mean costs were obtained by bias corrected and accelerated bootstrapping (2000 replications) [21]. Differences in costs and differences in ankle sprain recurrences were compared against the cheapest intervention (neuromuscular training) in a cost-effectiveness ratio, which estimated for each other condition the additional cost to prevent one ankle sprain recurrence. Confidence intervals for the cost-effectiveness ratio were calculated with bootstrapping, using the bias-corrected percentile method with 5000 replications. Uncertainty regarding the estimate of this ratio was expressed on a cost-effectiveness plane.

## Discussion

The ABrCt is the first randomized controlled trial to directly compare the secondary preventive effect of the combined use of braces and neuromuscular training, against the use of either braces or neuromuscular training as separate secondary preventive measures.

### Recruitment

Web-based recruitment was very successful. Between april and december 2010 356 participants were recruited. Due to logistical problems intervention packages were never received by 28 participants. These 28 participants had to be excluded from the study. Therefore, an additional 28 participants were recruited in april and may 2011. This resulted in a final total of 356 included participants.

One of the drawbacks of this method of inclusion is that there is no control over the content of the provided usual care. Although ruling clinical guidelines are considered usual care, this does not necessarily mean that caregivers are actually following these guidelines. Inclusion of participants through a limited number of controlled (para-) medical caregivers would have decreased this problem. However, inclusion through such channels is problematic and in our experience results in lower inclusion rates than expected. Even so, in the current study we are looking for patients treated by a variety of (para-)medical caregivers. Meaning a relatively large number of different caregivers would need to be found, informed on the study, and controlled as to their given treatment. Looking at the required number of participants this would prove an undoable and unrealistic undertaking. Moreover, the proposed study is on the effectiveness of secondary preventive measures (e.g. extended treatment options) that are being applied after usual care. Full control over usual care and its content would have hampered external validity of this study. Finally, when inclusion would have been done by the caregiver this would mean that randomization would have needed to take place at the level of the caregiver. Such a clustered design would have further complicated the study design.

### Co-intervention

Two Dutch medical guidelines explicitly describe the use of neuromuscular training and sports braces for the treatment of acute lateral ankle sprains. These are the guidelines of the Royal Dutch Physiotherapy Association (KNGF) and of the Dutch Association of Sports and Exercise Medicine (VSG). In the KNGF guideline neuromuscular training is mentioned as part of the primary treatment from 11 days to 12 weeks after sustaining an ankle sprain. Only in case of functional ankle instability neuromuscular training (on a balance board) is advised explicitly. A brace is advised in case of insufficient neuromuscular control. It is repeatedly advised to decrease brace use as soon as possible. Furthermore athletes are advised not to wear a brace during training, but only during matches. In the VSG guideline neuromuscular training is advised from day 5 after sustaining an ankle sprain (grade 1). The use of a brace after sport resumption is advised during 3 to 6 weeks depending on the severity of the sprain.

Taking into account the ruling KNGF and VSG guidelines, co-interventions through inclusion of similar interventions in the regular treatment protocol could have been possible. By registering the content of the provided care for the initial ankle sprain, for which participants were included in the study, we are able to correct for co-interventions. Nevertheless, the aim of this study was to evaluate the added value of neuromuscular training and brace use during sports on top of usual care as advised in these guidelines.

### Non-response

As stated, participants were recruited through calls placed on the internet. Interested participants completed a contact form on the study website, after which they were contacted by telephone to see if they were eligible for participation. As such it is unknown what percentage of the total eligible population was actually interested in participation. Albeit, the non-response after being contacted was low. Only a small percentage (2) didn't return the informed consent and baseline questionnaire after being informed on the study.

### Impact of results

This study expects to identify the most effective and cost-efficient secondary preventive measure for ankle sprains. The study results could lead to changes in the clinical guidelines on the prevention of ankle sprains, and they will become available in 2012.

## Competing interests

DJO Europe kindly provided the Aircast A60 Ankle Support braces and €5,000 of additional funding. The Rock balance boards were available from the previous study by our group (2Bfit study), provided by Avanco, Sweden. The authors declare that they have no competing interests.

## Authors' contributions

EALMV conceived of the idea and obtained funding for the study. EALMV and KWJ developed the intervention, developed the design of this trial, and KWJ recruited participants. WvM provided advice on the study design and contributed to the content of the article. KWJ is the study investigator, was responsible for data acquisition, and wrote the article. All authors read and approved the final manuscript.

## Authors' information

KWJ practises as a sports physician in the Netherlands currently at the Sports Medicine Centre Jeroen Bosch Hospital. He is also employed as junior researcher at the Department of Public and Occupational Health of the VU University Medical Center and the EMGO Institute in Amsterdam. This RCT will be the main study for his thesis. EALMV is human movement scientist and epidemiologist. He is employed as senior researcher at the Department of Public and Occupational Health of the VU University Medical Center and the EMGO Institute in Amsterdam, within the department's research theme 'Sports, Lifestyle and Health'. WvM is Head Department of Department of Public and Occupational Health of the VU University Medical Centre and Co-Director EMGO+ Institute of the VU University Medical Centre.

## Funding

The study is funded by the Netherlands Organization for Health Research and Development (ZonMW).

## Pre-publication history

The pre-publication history for this paper can be accessed here:

http://www.biomedcentral.com/1471-2474/12/210/prepub
